# Combined Microbiome and Metabolomic Analyses Reveal That Fine-Root Invasion of *Rhododendron auriculatum* Sapling Enhances Microbial Decomposition of *Sphagnum palustre* L.

**DOI:** 10.3390/microorganisms14051141

**Published:** 2026-05-17

**Authors:** Qiuxia Xiang, Guijun Bu, Xiaorong Tang, Changwu Shi, Bing Xiong, Lin Wu, Jia Xiong

**Affiliations:** Hubei Key Laboratory of Biological Resources Protection and Utilization, Hubei Minzu University, Enshi 445000, China; 202430467@hbmzu.edu.cn (Q.X.); 202210819@hbmzu.edu.cn (X.T.); 202211989@hbmzu.edu.cn (C.S.); 202212091@hbmzu.edu.cn (B.X.); wulin20054557@163.com (L.W.); xiongjia18@mails.ucas.ac.cn (J.X.)

**Keywords:** *Rhododendron auriculatum* Hemsl., *Sphagnum*, phenolics, decomposition, 16S rRNA, ITS region analysis

## Abstract

Phenolics in *Sphagnum* can inhibit its microbial decomposition. Climate warming and drainage have driven vascular plants, such as Ericaceae, to expand into *Sphagnum*-dominated peatland. However, the impact of fine root invasion by *Rhododendron auriculatum* Hemsl. on *Sphagnum* decomposition and changes in phenolic compounds remains unclear. This study compared *Sphagnum* decomposition in a *Sphagnum palustre* L.-dominated peatland and an *R. auriculatum* (Sapling)–*S. palustre* peatland by examining the microscopic structure of *S. palustre* and microbial community composition. Decomposition was higher in the *R. auriculatum–S. palustre* peatland. On this site, bacterial metabolic types such as aerobic chemoheterotrophy and chemoheterotrophy had higher relative abundances, as did fungal trophic modes, including those with combined ectomycorrhizal, ericoid mycorrhizal, and saprotrophic functions. Acid phosphatase, laccase, total nitrogen (TN), C/N ratio (C:N), and pH differed significantly across decomposition stages. Microbial communities are affected by physicochemical factors and enzyme activities. Untargeted metabolomics revealed more downregulated than upregulated phenolics, cinnamic acids, and tannins, indicating loss of phenolic compounds. In summary, *R. auriculatum* fine root invasion altered enzyme activities and physicochemical properties, driving the restructuring of bacterial and fungal trophic modes and accelerating *S. palustre* cell wall and hyaline cell decomposition.

## 1. Introduction

*Sphagnum*-dominated peatlands are extensively distributed across North America, ranging from the subarctic regions to the southern margin of the cold temperate zone [1]. In China, these peatlands are primarily located in the northeastern part of the country [2], as well as in several southern regions, including Niangniang Mountain in Guizhou Province [3], Dajiuhu in Hubei Province [4], and Qizimei Mountain and Taishanmiao in Enshi [5]. Previous studies on the Changbai Mountains have documented a range of peatland mosses, including *Aulacomnium palustre*, *Sphagnum fallax*, *S. magellanicum*, *S. capillifolium*, *Polytrichum juniperinum*, *S. fuscum*, and *S. palustre* [6]. Among these, *S. palustre* exhibits a broader distribution and serves as the dominant *Sphagnum* species in Dajiuhu, Hubei Province [4], as well as in Qizimei Mountain and Taishanmiao in Enshi [5]. *Sphagnum* is the dominant component species of *Sphagnum*-dominated peatlands and represents a group of mosses characterized by distinctive morphological, physiological, biochemical, and developmental traits [7,8]. *Sphagnum* comprises two specialized cell types: chlorophyllous cells and hyaline cells. The latter are dead cells lacking chloroplasts but functioning in water storage, conduction, and structural support, thereby forming a fundamental component of *Sphagnum* stem and leaf anatomy [9,10,11]. Notably, sphagnic acid (p-hydroxy-β-(carboxymethyl) cinnamic acid) is a characteristic compound associated with these hyaline cells [11]. In addition, the cell walls of hyaline cells consist of cellulose microfibrils embedded within an amorphous network of phenolic polymers. This matter is widely recognized for its suppressive effects on microbial decomposition of *Sphagnum* tissues [12,13,14]. In recent decades, global warming and anthropogenic disturbances such as drainage have driven extensive shrub encroachment into peatlands, including species from the Ericaceae, as well as *Rhamnus crenata* Sieb. et Zucc. and *Camellia sinensis* (L.) O. Kuntze [3,15,16,17]. A study on shrub invasion in grasslands reported that approximately fifty percent of soil phenolic compounds were consumed following encroachment [18]. In contrast, other research has shown that invasion by ericaceous plants can lead to an increase in phenolic content within peatlands [19]. Given these contrasting observations, how *R. auriculatum* invasion into *Sphagnum* peatlands influences phenolic compounds and the microbial communities involved in *Sphagnum* decomposition remains to be studied further.

Acid-tolerant anaerobic bacteria and saprotrophic fungi play important roles in *Sphagnum* decomposition. Key contributors include members of the Acidobacteriota (original designation: Acidobacteria), many of which are capable of degrading complex organic substrates [12,20,21]. Genomic evidence indicates that Acidobacteriota encode a diverse array of carbohydrate-active enzymes involved in the degradation of xylan, cellulose, hemicellulose, and pectin and also participate in the turnover of amino acids, alcohols, and intermediary metabolites [20,22]. Pseudomonadota (original designation: Proteobacteria) are known to degrade phenolic compounds and assimilate uronic acids, whereas Actinobacteria specialize in the decomposition of recalcitrant polymers. Planctomycetes contribute to the degradation of heteropolysaccharides and preferentially occupy anoxic peat microhabitats, exhibiting strong adaptation to oligotrophic conditions and thereby facilitating late-stage litter transformation [8]. Verrucomicrobia are among the most active taxa involved in the degradation of complex polysaccharides, such as substrates derived from fucose, rhamnose, and hemicellulose [23,24]. Within the fungal domain, certain Ascomycota taxa degrade cellulose and tannins, disrupting plant cell wall components, while most Basidiomycota have trophic modes as saprotrophs that decompose cellulose and insoluble phenolic compounds, often preferentially removing polyphenolic matrices from cell walls [12]. In addition, members of the Mucoromycota are capable of degrading selected recalcitrant aromatic compounds [25]. Despite these advances, it remains unclear whether microbial associations in the fine roots of *R. auriculatum* possess the capacity to decompose the recalcitrant organic matter of *Sphagnum*.

The metabolomic composition of host plants is strongly shaped by diverse microbial associations, while microbial decomposition of *Sphagnum* may, in turn, alter its endogenous metabolites [26]. Studies across different *Sphagnum* microhabitats have demonstrated that *Sporichthya* is significantly negatively correlated with icosadienoic acid and cannabidiol and also shows significant associations with other metabolites [27]. In *Ginkgo biloba*, the abundance of *Staphylococcus* is closely correlated with levels of quercetin and total flavonoids [28]. Similarly, in *Cynomorium songaricum*, the key compound gallic acid exhibits significant correlations with the most dominant endophytic fungi [29]. During the co-evolution of microbial associations and their host plants, microorganisms not only participate in the biosynthesis of plant secondary metabolites but may also directly produce compounds structurally analogous to those synthesized by the host [30]. These findings highlight the necessity of understanding how microbial decomposition of *Sphagnum* influences its metabolite composition. In recent years, high-throughput sequencing approaches based on 16S rRNA gene and ITS region analysis, coupled with functional prediction tools such as FAPROTAX, PICRUSt, and FUNGuild, have enabled comprehensive characterization of microbial associations’ composition, diversity, and trophic mode potential, thereby facilitating deeper insights into plant and microbe interactions [27,31,32,33,34]. Nevertheless, the effects of microbial associations associated with the fine roots of *R. auriculatum* on the metabolome composition of *Sphagnum* at different stages of decomposition remain largely unexplored.

In this study, we hypothesize that the invasion of fine roots of *Rhododendron auriculatum* enhances the abundance of saprophytic and mycorrhizal fungi involved in *Sphagnum palustre* L. decomposition and that the decomposition of *Sphagnum* may lead to the loss of phenolics, cinnamic acids, and tannins. To test this hypothesis, we employed 16S rRNA gene and ITS region sequencing techniques to analyze the microbial community composition and diversity in fine roots of *R. auriculatum* and in *S. palustre* litter at different stages of decomposition and to predict the functional genes of the microbial communities. Untargeted metabolomics was further applied to quantify variation in phenolic compounds, cinnamic acid derivatives, and tannins across decomposition gradients. Spearman’s correlation analysis was used to assess associations between microbial taxa and these metabolite classes. In summary, this study aims to elucidate how fine root encroachment by *R. auriculatum* influences bacterial and fungal contributions to *S. palustre* decomposition, thereby providing a theoretical framework for the conservation of subtropical subalpine *S. palustre*-dominated peatland.

## 2. Materials and Methods

### 2.1. Study Site

**Figure 1:**
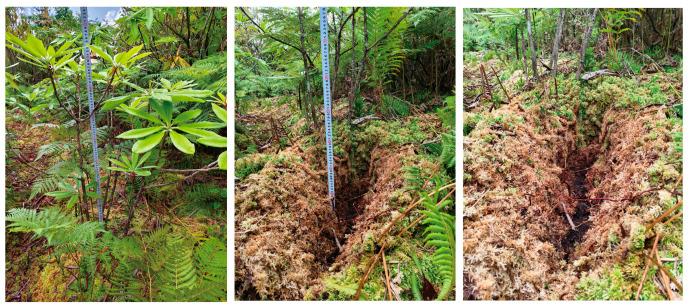
Field photograph showing the current condition of the *Rhododendron auriculatum* and *S. palustre* peatland plot.

Samples were collected from a peatland situated at Tai Shanmiao (TSM; 30°14′ N, 109°8′ E; 1932.7–1933.4 m above sea level) in the Enshi Tujia and Miao Autonomous Prefecture, Hubei Province, China. The study area has a subtropical monsoon humid climate. The landform of the study plot is characterized as a depression, representing a rain-fed, ombrotrophic *Sphagnum* peatland. The water table depths are −18.6 cm, −20.1 cm, and −21 cm. Figure 1 presents photographs of the *S. palustre* sampling core and an overview of the study site landscape.

### 2.2. Field Survey and Sampling

The study plot measured twenty meters by fifteen meters and was dominated by *S. palustre*, *Rhododendron auriculatum*, and *Pteridium aquilinum*. Within *S. palustre*-dominated peatlands and the *R. auriculatum* (Sapling)–*S. palustre* peatlands, *S. palustre* samples and fine roots of *R. auriculatum* were collected. Prior to sampling, surface debris was carefully removed, and samples were obtained from a depth of approximately fifteen to twenty centimeters. We first observed the color changes of *S. palustre* during the decomposition process. Based on these color differences, we then examined the micro morphological structures of *S. palustre* with different colors using a scanning electron microscope (SEM; JEOL, JSM 6510LV, Japan). According to micro morphological indicators, including the integrity of cell walls and hyaline cells, as well as the degree of cellular structural disruption (detailed in Section 3.1), we classified the decomposition degree of *S. palustre* into four levels. In the *R. auriculatum–S. palustre* peatland, green *Sphagnum* (approximately 3–4 cm in length) was classified as undecomposed (W); yellowish brown *S. palustre* as slightly decomposed (D); brownish gray *S. palustre* as moderately decomposed (Z); and dark brown *S. palustre* as highly decomposed (G). The lengths of D, Z, and G were all approximately 4–5 cm. In the *S. palustre*-dominated peatland, following the same criteria, samples were collected and designated as CKW for undecomposed, CKD for slightly decomposed, and CKZ for moderately decomposed. Samples designated for 16S rRNA gene sequencing, ITS sequencing, and metabolomic analyses were transported on dry ice to Shanghai Majorbio Bio-Pharm Technology Co., Ltd. (Shanghai, China) for subsequent processing. Samples for enzyme activity assays were stored at −80 °C until analysis.

### 2.3. Enzyme Activity Assays and Physicochemical Factors

β-glucosidase, acid phosphatase, phenol oxidase, and laccase were extracted using commercial assay kits, and their absorbance was measured at 405 nm [35], 400 nm [36], 450 nm, and 420 nm [37], respectively, using a microplate reader (Multiskan FC, Thermo Fisher Scientific, Waltham, MA, USA). Total carbon (TC) and total nitrogen (TN) were determined using a vario MICRO cube elemental analyzer (Elementar, Langenselbold, Germany). The pH (*Sphagnum* (W)/deionized water (V), 1:20) was measured using a pH meter (Mettler Toledo FiveEasy, Greifensee, Switzerland).

### 2.4. 16S rRNA Gene and ITS Region Sequencing Analysis

Genomic DNA was extracted using the TruSeq™ DNA Sample Prep Kit (Illumina, San Diego, CA, USA) according to the manufacturer’s instructions. Briefly, 0.5 g of sample from each replicate was used for DNA extraction. The V3–V4 region of the bacterial 16S rRNA gene was amplified using primers 338F (5′-ACTCCTACGGGAGGCAGCAG-3′) and 806R (5′-GGACTACHVGGGTWTCTAAT-3′) [38]. The fungal internal transcribed spacer (ITS) region was amplified using primers ITS1F (5′-CTTGGTCATTTAGAGGAAGTAA-3′) and ITS2R (5′-GCTGCGTTCTTCATCGATGC-3′) [27]. PCR amplification was performed under the following thermal cycling conditions: initial denaturation at 95 °C for 3 min, followed by 30 cycles of denaturation at 95 °C for 30 s, annealing at 55 °C for 30 s, and extension at 72 °C for 45 s, with a final extension at 72 °C for 10 min. Primers used in each reaction contained unique error-correcting barcodes of varying lengths. Each PCR reaction was carried out in a total volume of 20 μL, consisting of 10 μL of 2× Pro Taq, 0.8 μL of 5 μM forward primer, 0.8 μL of 5 μM reverse primer, and template DNA at a concentration of 10 ng/μL. To minimize amplification bias, each sample was amplified in triplicate, and the resulting products were pooled. All PCR reactions were performed using an ABI GeneAmp^®^ 9700 thermal cycler (Thermo Fisher Scientific, Waltham, MA, USA). PCR products from the same sample were combined and verified by electrophoresis on a 2% agarose gel, followed by purification using AMPure^®^ PB beads (Beckman Coulter, Brea, CA, USA). The purified amplicons were then pooled in equimolar concentrations and sequenced according to standard protocols at Shanghai Majorbio Bio-Pharm Technology Co., Ltd. (Shanghai, China). Finally, functional profiles of bacterial and fungal communities were predicted using FAPROTAX [34], PICRUSt2 [27], and FUNGuild [33].

### 2.5. Metabolomics Analysis

Approximately 50 mg of sample was placed into a 2 mL centrifuge tube, followed by the addition of 6 mm grinding beads and 400 μL of extraction solvent (methanol/water, 4:1, *v*/*v*) containing four internal standards, including L-2-chlorophenylalanine (0.02 mg/mL). Samples were homogenized using a cryogenic tissue grinder for 6 min (−10 °C, 50 Hz), followed by ultrasonic extraction at low temperature for 30 min (5 °C, 40 kHz). The extracts were then incubated at −20 °C for 30 min and centrifuged at 13,000× *g* for 15 min at 4 °C. The supernatant was transferred into autosampler vials with inserts for subsequent analysis. In addition, 20 μL of supernatant from each sample was pooled to generate a quality control (QC) sample.

Liquid chromatography–mass spectrometry (LC–MS) analysis was performed on a UHPLC–Orbitrap MS system (UHPLC–Exploris 480; Thermo Fisher Scientific). Chromatographic separation was achieved using an ACQUITY UPLC HSS T3 column (100 mm × 2.1 mm i.d., 1.8 μm; Waters, Milford, CT, USA). The mobile phase consisted of solvent A (95% water and 5% acetonitrile with 0.1% formic acid) and solvent B (47.5% acetonitrile, 47.5% isopropanol, and 5% water with 0.1% formic acid). The injection volume was 3 μL, and the column temperature was maintained at 40 °C. Mass spectrometric data were acquired using electrospray ionization in both positive and negative ion modes. Differential metabolites were identified based on variable importance in projection (VIP) ≥ 1 and *p* < 0.05 [39,40].

### 2.6. Statistical Analysis and Visualization

One-way analysis of variance (ANOVA) was used to assess differences in enzyme activities among groups. Principal coordinates analysis (PCoA) was used to analyze the differences between taxa. Spearman’s rank correlation analysis was applied to examine associations between variables. All statistical analyses and data visualizations were conducted on the Majorbio Cloud Platform (www.majorbio.com).

## 3. Results

### 3.1. Structural Changes and Differences in Enzyme Activity of S. palustre

**Figure 2:**
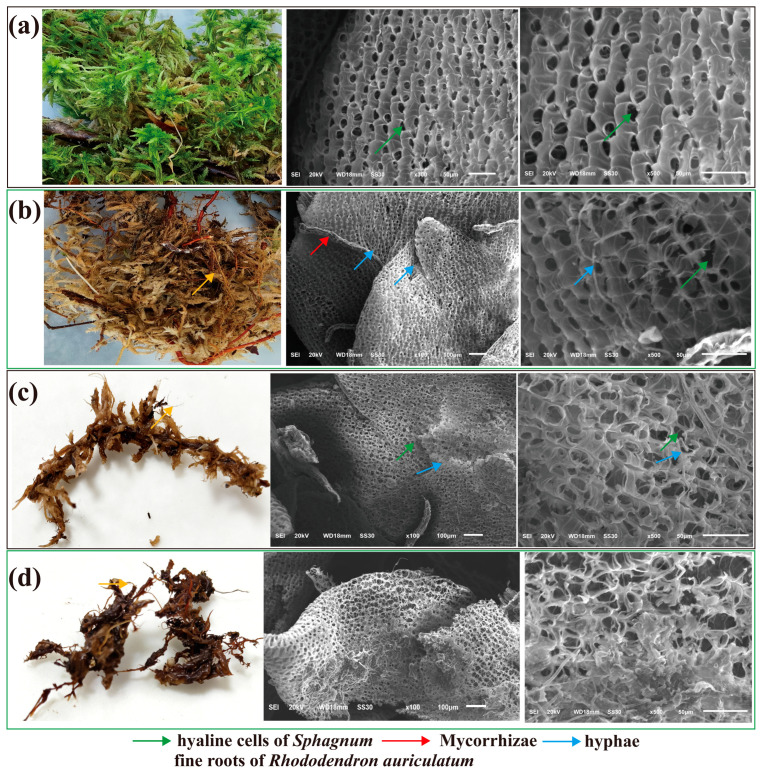
Morphological changes in *Sphagnum* hyaline cells. Undecomposed *S. palustre* (**a**), slightly decomposed *S. palustre* and the fine roots of *R. auriculatum* (**b**), moderately decomposed *S. palustre* (**c**), and highly decomposed *S. palustre* (**d**).

Scanning electron microscopy revealed clear structural differences across *Sphagnum* decomposition stages in the *R. auriculatum–S. palustre* peatland. Undecomposed *Sphagnum* (W) maintained intact structural integrity. In the slightly decomposed stage (D), fungal hyphae were attached to the surface, and both leaf tissue and hyaline cells of *S. palustre* showed initial degradation. In the moderately decomposed stage (Z), hyaline cells became disrupted and irregular shapes. In the highly decomposed stage (G), the hyaline cells are completely decomposed (Figure 2a–d). In contrast, in the *S. palustre*-dominated peatland, decomposition levels at the undecomposed (CKW), slightly decomposed (CKD), and moderately decomposed (CKZ) stages were consistently lower than those in the *R. auriculatum–S. palustre* peatland (Figure S7).

**Figure 3:**
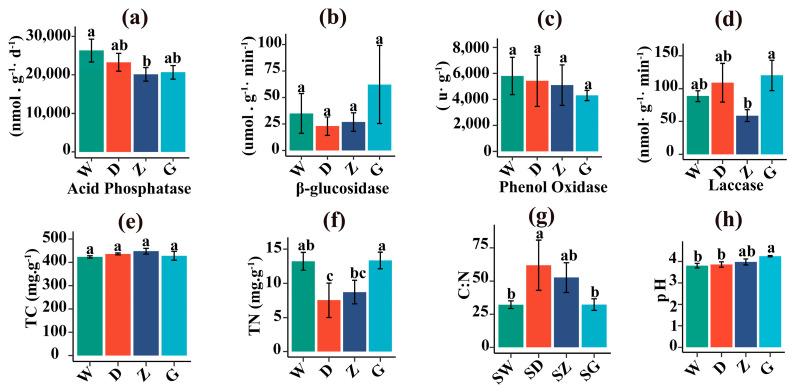
Differences in enzyme activities of *S. palustre*. (**a**) Acid phosphatase, (**b**) β-glucosidase, (**c**) phenol oxidase, (**d**) laccase, (**e**) total carbon (TC), (**f**) total nitrogen (TN), (**g**) C/N ratio (C:N), and (**h**) pH. Different lowercase letters indicate significant differences among treatments at *p* < 0.05.

Figure 3a–h presents the comparative analysis of enzyme activities and physicochemical properties. Acid phosphatase activity exhibited an overall decreasing trend, with significantly lower activity in Z compared to W (Figure 3a). Laccase activity increased from W to D, declined from D to Z, and then increased significantly from Z to G (Figure 3d). In contrast, no significant differences were observed for β-glucosidase and phenol oxidase activities across decomposition stages (Figure 3b,c). Total nitrogen (TN) content significantly decreased from W to D, followed by a significant increase from D to G (*p* < 0.05). The carbon to nitrogen ratio (C:N) exhibited a trend opposite to that of TN, with a significant increase from W to D and a significant decrease from D to G (*p* < 0.05) (Figure 3f,g). The pH value increased significantly from W to G and remained below 4.3 on average throughout the decomposition gradient (*p* < 0.05) (Figure 3h). In contrast, total carbon (TC) content did not show any significant variation across the different decomposition stages (Figure 3e).

### 3.2. Analysis of Bacterial and Fungal Diversity in S. palustre and Fine Roots of R. auriculatum

A total of 1,267,026 and 1,145,626 high-quality 16S rRNA gene and ITS sequences were obtained from *R. auriculatum* fine roots and *S. palustre*, respectively (Tables S1 and S2). A total of 7898 bacterial ASVs and 1425 fungal ASVs were retained for subsequent analyses. Bacterial sequences were assigned to 35 phyla, whereas fungal sequences were classified into 10 phyla. Across all samples, 297 bacterial families and 202 fungal families were identified (Table S3).

The diversity of bacterial and fungal association in *S. palustre* and *R. auriculatum* fine roots was analyzed. Alpha diversity analyses revealed no significant differences in bacterial Chao1 and Shannon indices (Figure S1a,c). The fungal Chao1 index was lowest in the fine roots of *R. auriculatum* and decreased significantly from stage W to stage G (Figure S1b), while the Shannon index showed no significant variation (Figure S1d).

We employed Principal Coordinates Analysis (PCoA) to evaluate differences in bacterial and fungal community structures. For the bacterial communities, the first two principal coordinates (PC1 and PC2) explained 38.56% of the total variation, while for the fungal communities, they accounted for 40.59% of the total variation. These results indicate that the PCoA model captures a substantial portion of the community variation, demonstrating its strong explanatory power (Figure S1e,f).

**Figure 4:**
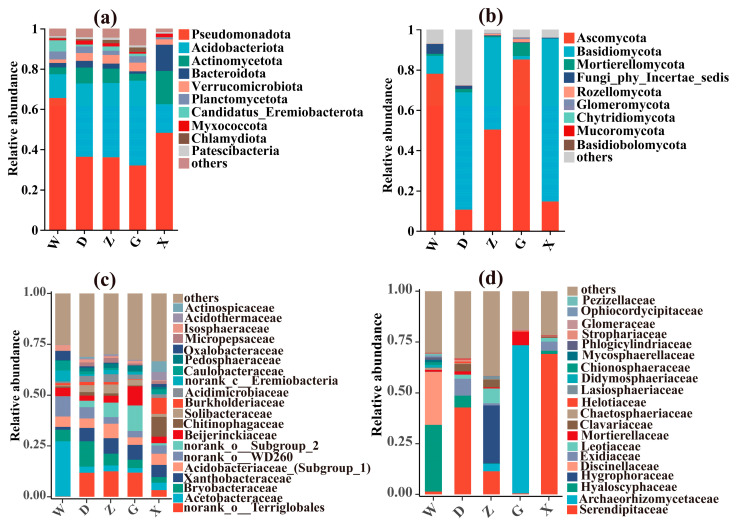
Analysis of dominant bacterial and fungal taxa in *S. palustre* and *R. auriculatum* fine roots. (**a**) Bacterial communities at the phylum level; (**b**) fungal communities at the phylum level; (**c**) bacterial communities at the family level; (**d**) fungal communities at the family level.

This study analyzed the differences in bacterial and fungal communities at the phylum level between *S. palustre*-dominated peatland and *R. auriculatum–S. palustre* peatland (Figure 4 and Figure S2). Among bacteria (Figure 4a and Figure S2a), the relative abundances of Pseudomonadota in CKW, CKD, and CKZ were 40.63%, 22.41%, and 33.12%, respectively, which arte lower than the corresponding values in W (65.51%), D (36.35%), and Z (36.1%). Conversely, Acidobacteriota exhibited higher relative abundance in CKD (44.95%) and CKZ (43.46%) compared to D (36.33%) and Z (36.83%). Within the fungal communities (Figure 4b and Figure S2b), Ascomycota dominated in CKD (6.02%) and CKZ (32.61%), whereas Basidiomycota was predominant in D (58.26%). In Z, the two phyla exhibited co-dominance, with relative abundances of 45.79% for Basidiomycota and 50.31% for Ascomycota. Additionally, the relative abundances of Actinobacteriota, Bacteroidota, and Basidiomycota were higher in the fine roots of *R. auriculatum* (X) than in *S. palustre*.

To further investigate microbial succession and community differences, we focused on the 20 most abundant taxa at the family level. In CKW, the dominant bacterial taxa included an uncharacterized group within the order Terriglobales (original designation: norank_o__Terriglobales), Bryobacteraceae, and Acetobacteraceae, while in W, Acetobacteraceae prevailed. The uncharacterized group within the order Terriglobales was the dominant bacterial group in CKD (28%), whereas in D, Bryobacteraceae (12.59%) and the uncharacterized group within the order Terriglobales (11.79%) were the main bacterial taxa (Figure 4c and Figure S2c). In X, dominant bacterial groups included Xanthobacteraceae, Acidobacteriaceae (Subgroup_1), Chitinophagaceae, and Burkholderiaceae (Figure 4c). Among fungi, CKW was dominated by Hyaloscyphaceae, whereas W showed co-dominance of Hyaloscyphaceae and Discinellaceae. In CKD, Hyaloscyphaceae remained dominant, while D was dominated by Serendipitaceae, Hyaloscyphaceae, and Exidiaceae. CKZ was primarily dominated by Archaeorhizomycetaceae and Hygrophoraceae, whereas Z was co-dominated by Hygrophoraceae, Serendipitaceae, and Leotiaceae. Notably, Archaeorhizomycetaceae accounted for 72.88% of the fungal community in G, and Serendipitaceae represented 69.1% in X (Figure 4d and Figure S2d). Additionally, the relative abundances of Exidiaceae, Leotiaceae, and Helotiaceae increased from W to D and remained relatively high in X (Figure S3a–c).

### 3.3. Functional Annotation of Bacteria and Fungi

Bacterial metabolic types and fungal trophic modes were analyzed using FAPROTAX and FUNGuild, including only taxa with relative abundance > 0.5% (Figures S2d,e and S4a,b). In comparisons between the large *S. palustre*-dominated peatland and the *R. auriculatum–S. palustre peatland*, the relative abundances of bacteria with chemoheterotrophic and aerobic chemoheterotrophic metabolic types were lower in CKD (33.54%, 30.9%) than in D (35.44%, 32.6%), and lower in CKZ (30.59%, 27.71%) than in Z (33.68%, 31.88%) (Figures S2e and S4b). Fungal trophic modes, including orchid mycorrhizal and bryophyte parasite–ectomycorrhizal–ericoid mycorrhizal–undefined saprotroph–wood saprotroph, were also lower in CKD and CKZ than in D (33.09%, 2.12% ) and Z (8.14%, 7.21%) (Figures S2f and S4a). In CKD, bacterial cellulolysis and fungal trophic modes such as fungal parasite–undefined saprotroph and ectomycorrhizal–endophyte–ericoid mycorrhizal–litter saprotroph–orchid mycorrhizal were lower than in D (Figures S2d,e and S4a,b).

**Figure 5:**
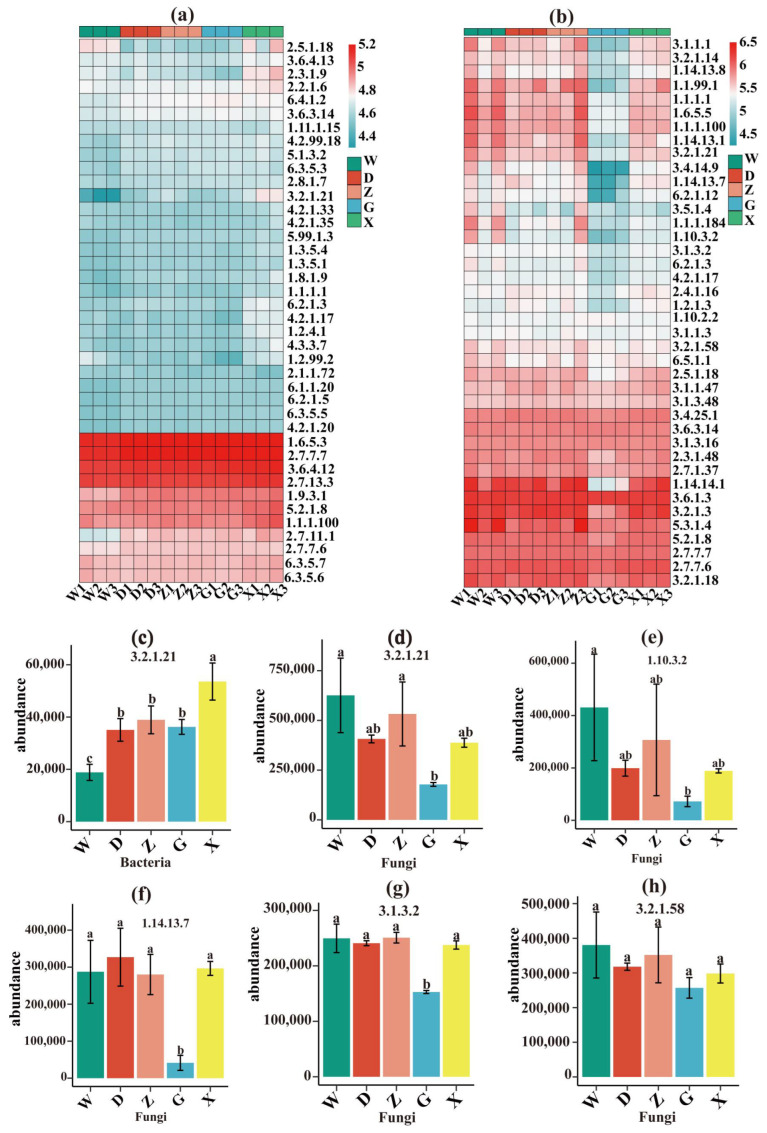
Annotation of genes encoding enzymes in bacteria and fungi. (**a**) Heatmap of genes encoding enzymes in bacteria, (**b**) heatmap of genes encoding enzymes in fungi, (**c**) bacterial β-glucosidase genes, (**d**) fungal β-glucosidase genes, (**e**) fungal laccase genes, (**f**) fungal phenol 2-monooxygenase genes, (**g**) fungal acid phosphatase genes, (**h**) fungal glucan 1,3-β-glucosidase genes. Different lowercase letters indicate significant differences among treatments at *p* < 0.05.

PICRUSt2 predicted the top 40 enzyme-coding genes in bacteria and fungi (Figure 5a,b). Both bacteria and fungi were annotated with β-glucosidase (EC 3.2.1.21). Fungi additionally contained laccase (EC 1.10.3.2), phenol 2-monooxygenase (EC 1.14.13.7), acid phosphatase (EC 3.1.3.2), and glucan 1,3-β-glucosidase (EC 3.2.1.58). Bacterial β-glucosidase increased significantly from W to X (Figure 5c). Fungal β-glucosidase decreased significantly from W to G (Figure 5d). Laccase abundance in fungi declined from W to G (Figure 5e). Phenol 2-monooxygenase was highest in D and lowest in G (Figure 5f). Acid phosphatase peaked in Z and was lowest in G (Figure 5g). No significant differences were found for glucan 1,3-β-glucosidase (Figure 5h). These enzymes can degrade *Sphagnum* cell walls, phenolic compounds, and other organic matter.

### 3.4. Differences in Phenolics, Cinnamic Acid Derivatives, and Tannins

We analyzed the differential metabolites, including phenolics, cinnamic acids, and tannins, in *S. palustre* from decomposition stages W, D, Z, and G (VIP ≥ 1, *p* < 0.05). Between W and D, a total of 13 differential metabolites were identified, of which 8 were upregulated and 5 were downregulated (Figure S5a). Between W and Z, 19 differential metabolites were detected, with 10 upregulated and 9 downregulated (Figure S5b). Between W and G, 18 differential metabolites were identified, including 8 upregulated and 10 downregulated (Figure S5c). Between Z and D, one metabolite was downregulated (Figure S5d). Between D and G, five differential metabolites were detected, with four upregulated and one downregulated (Figure S5e). Between Z and G, one metabolite was upregulated (Figure S5f).

In addition, a total of nine common differential metabolites were shared among the pairwise comparisons of between W and D, between W and Z, and between W and D. Among these, the number of downregulated metabolites was greater than the number of upregulated ones (Figure S6a, Table S4).

### 3.5. Correlation Analysis Between Enzyme Activities and Microbial Association

**Figure 6:**
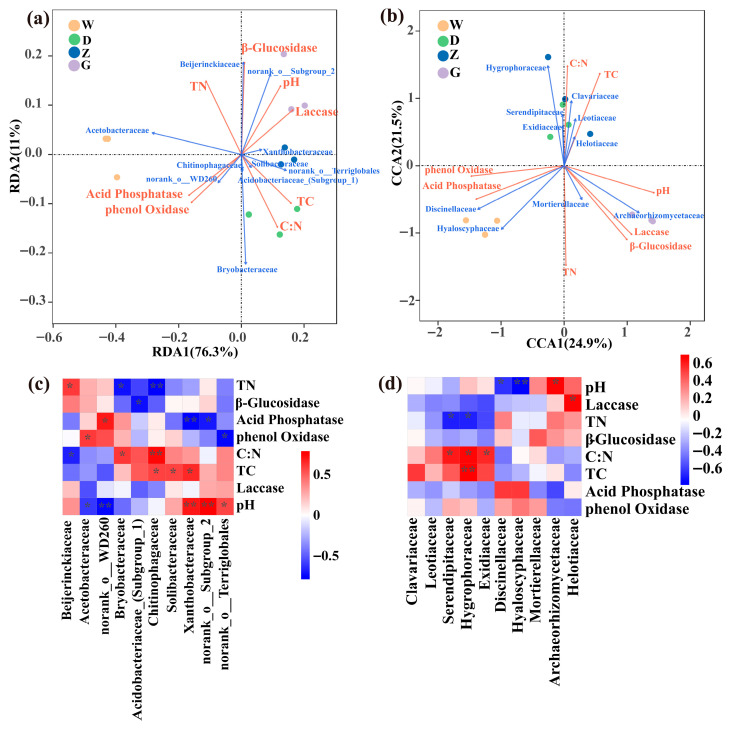
Correlation analysis of microorganisms with enzyme activities and physicochemical fac tors: (**a**) redundancy analysis (RDA) of bacteria, (**b**) canonical correspondence analysis (CCA) of fungi, (**c**) correlation analysis between bacteria and enzyme activities, (**d**) correlation analysis between fungi and enzyme activities. The significance levels of correlation coefficients were marked on each plot by asterisks (*p* < 0.01, **; *p* < 0.05, *).

Redundancy analysis (RDA) and canonical correspondence analysis (CCA) were applied to evaluate the relationships of enzyme activities and physicochemical factors with bacterial and fungal associations, respectively (Figure 6a,b). In bacterial families, TN and pH were positively correlated with β-glucosidase and laccase activities but showed weaker correlations with acid phosphatase and phenol oxidase activities and were negatively correlated with TC and the C:N ratio. The C:N ratio was positively correlated with TC, acid phosphatase, and phenol oxidase, but negatively correlated with β-glucosidase and laccase (Figure 6a). For fungal families, the correlation results were consistent with those for bacterial families, except that the C:N ratio was negatively correlated with acid phosphatase and phenol oxidase (Figure 6b).

Spearman correlation analysis was performed to examine the relationships between the top ten abundant bacterial and fungal families and enzyme activities. The bacterial results showed that Acetobacteraceae was significantly negatively correlated with pH (*p* < 0.05), whereas the uncharacterized group within the order Terriglobales was significantly positively correlated with pH (*p* < 0.05) (Figure 6c). Among the fungal taxa (Figure 6d), Helotiaceae exhibited a significant positive correlation with laccase activity (*p* < 0.05). Serendipitaceae and Hygrophoraceae were significantly positively correlated with the C:N ratio (*p* < 0.05) and significantly negatively correlated with TN (*p* < 0.05). Archaeorhizomycetaceae was significantly positively correlated with pH (*p* < 0.05).

### 3.6. Correlation Analysis Between Microbial Association and Metabolomic Profiles

**Figure 7:**
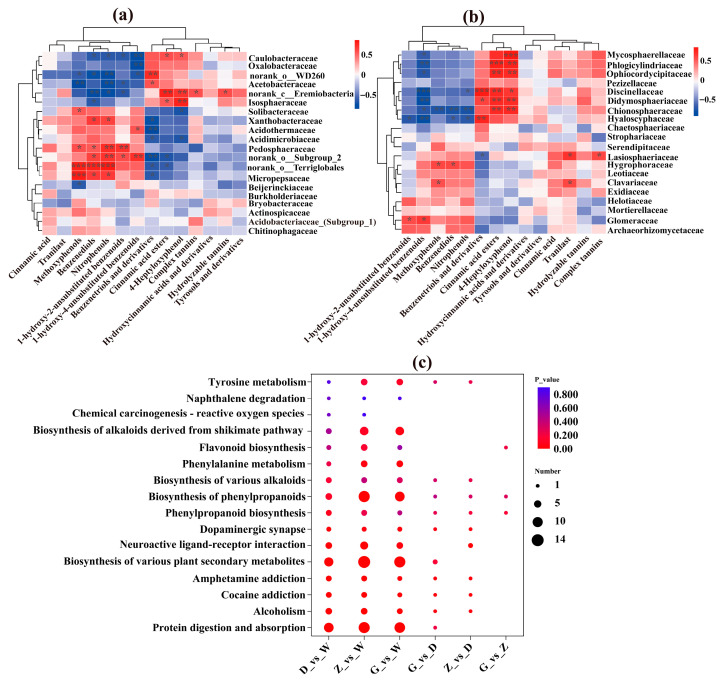
Correlation analysis among bacteria, fungi, and metabolites. (**a**) Spearman correlation analysis between bacteria and metabolites; (**b**) Spearman correlation analysis between fungi and metabolites; (**c**) KEGG pathway enrichment analysis. The significance levels of correlation coefficients were marked on each plot by asterisks (*p* < 0.001, ***; *p* < 0.01, **; *p* < 0.05, *).

Spearman correlation analysis revealed distinct associations between bacterial taxa and metabolite classes (Figure 7a). Acetobacteraceae showed a significant positive correlation with benzenetriols and their derivatives. Xanthobacteraceae was significantly and positively correlated with benzenediols and nitrophenols (0.01 < *p* < 0.05). An uncharacterized group within the order Terriglobales exhibited extremely strong positive correlations with methoxyphenols, benzenediols, and nitrophenols (*p* < 0.001) (Figure 7a). Similarly, norank_o_Subgroup 2 (belonging to the candidate order Candidatus Acidoferrales) was significantly positively correlated with benzenediols and 1-hydroxy-2-unsubstituted benzenoids (0.01 < *p* < 0.05) and showed strong positive correlations with nitrophenols and 1-hydroxy-4-unsubstituted benzenoids (0.001 < *p* < 0.01). In fungi (Figure 7b), Hygrophoraceae was significantly positively correlated with methoxyphenols and benzenediols (0.01 < *p* < 0.05).

KEGG pathway enrichment analysis indicated that pathways in D vs. W were significantly enriched in the biosynthesis of various plant secondary metabolites, whereas both Z vs. W and D vs. W were significantly enriched in the biosynthesis of various plant secondary metabolites and the biosynthesis of phenylpropanoids (Figure 7c).

## 4. Discussion

### 4.1. Bacteria and Fungi Associated with Fine Roots of R. auriculatum Synergistically Promote S. palustre Decomposition

A growing body of evidence has demonstrated that Serendipitaceae engage in diverse interactions with plants, including orchid mycorrhiza, ericoid mycorrhiza, and symbioses with bryophytes [41,42,43]. In addition, members of Serendipitaceae possess a large repertoire of genes associated with saprotrophic lifestyles and may acquire carbon through saprotrophic pathways [43]. Our results showed that *S. palustre* decomposition was enhanced in the *R. auriculatum*-*S. palustre* peatland compared with the *S. palustre*-dominant peatland (Figure 1). In D, Serendipitaceae was the dominant taxon, whereas other taxa collectively accounted for 88.02% in CKD (Figure 4d and Figure S2d). Following the invasion of *S. palustre* by fine roots of *R. auriculatum*, the dominance of Serendipitaceae in D may contribute to accelerated *S. palustre* decomposition. Bryobacteraceae and an uncharacterized group within the order Terriglobales were the dominant bacterial groups in D, whereas Xanthobacteraceae, Acidobacteriaceae (Subgroup_1), Chitinophagaceae, and Burkholderiaceae dominated in X. Notably, the relative abundance of Xanthobacteraceae increased progressively from W to Z (Figure 4c). *Sphagnum* is enriched in phenolic compounds, and its cell walls contain abundant cellulose and other heteropolysaccharides, which collectively confer resistance to microbial degradation [11]. Terriglobia, such as Bryobacteraceae and Acidobacteriaceae, are enriched in oligotrophic environments such as acidic soils and peatlands and are capable of utilizing and degrading a wide range of heteropolysaccharides, including plant-derived cellulose and microbial polysaccharides [44,45,46]. Some members of Xanthobacteraceae can grow on specialized substrates, including alkenes, halogenated aliphatic and aromatic compounds, terpenes, thiophenes, and polyaromatic compounds [47]. Burkholderiaceae comprises diverse microorganisms, including saprotrophs and plant pathogens, while chitinophagaceae are capable of degrading gelatin, cellulose, and chitin [48,49]. These findings are consistent with our results.

The relative abundance of bacteria involved in cellulolysis in D was higher than in CKD and increased progressively from W to Z (Figures S2e and S4c). Bacterial groups involved in cellulolysis, such as Actinobacteria and Pseudomonadota, can produce cellulases that degrade crystalline cellulose in plant cell walls [50,51]. Most saprotrophic fungi belong to Ascomycota and Basidiomycota and decompose plant litter and soil organic matter through their enzymatic systems [52,53]. In addition to orchid mycorrhizal being a dominant trophic mode in D, saprotrophic fungi were more abundant in D and X than in CKD (Figures S2f and S4a). This suggests that the invasion of *S. palustre* by fine roots of *R. auriculatum* may increase the abundance of saprotrophic fungi, thereby promoting *S. palustre* decomposition, which is consistent with our hypothesis.

### 4.2. Downregulation of Phenolics, Cinnamic Accids, and Tannins During Sphagnum Decomposition

Our study revealed a strong positive correlation between Xanthobacteraceae and benzenediols and nitrophenols (Figure 7a). Ge et al. reported that Xanthobacteraceae can generate phenolic intermediates during the degradation of polycyclic aromatic hydrocarbons (PAHs), which are subsequently oxidized and ultimately funneled into the tricarboxylic acid cycle [54]. In addition, certain species within Xanthobacteraceae are capable of utilizing a range of specialized substrates, including alkenes, halogenated aliphatic and aromatic compounds, terpenes, thiophenes, and polyaromatic compounds as sources of carbon and energy [47]. These findings suggest that Xanthobacteraceae possess the capacity for phenolic transformation, with the resulting phenolic intermediates potentially further degraded by other microbes. Although direct evidence for phenolic degradation by Hygrophoraceae is currently lacking, Agaricomycetes are known to degrade recalcitrant lignocellulosic substrates [55,56], providing a plausible functional explanation for the observed significant positive correlation between Hygrophoraceae and Methoxyphenols and Benzenediols (Figure 7b).

Using PICRUSt2, we predicted that fungi from both *S. palustre* and *R. auriculatum* fine roots harbor genes encoding laccase and phenol 2-monooxygenase (Figure 5e,f). Laccases are capable of degrading recalcitrant phenolic polymers [57,58], while phenol 2-monooxygenase is a redox enzyme that converts phenols to catechols, thereby facilitating phenolic decomposition [59,60]. This supports our findings that, relative to W, the total number of downregulated phenolic metabolites increased progressively in D_vs_W, Z_vs_W, and G_vs_W comparisons, with the total number of downregulated phenolic metabolites exceeding upregulated ones (Figures S5 and S6). Comparing trophic modes between *S. palustre* peatland and *R. auriculatum-S. palustre* peatland, we observed increased relative abundances of bryophyte parasite–ectomycorrhizal–ericoid mycorrhizal–undefined saprotroph–wood saprotroph and ectomycorrhizal–endophyte–ericoid mycorrhizal–litter saprotroph–orchid mycorrhizal from W to D, with these guilds also present in X (Figure S4e,f). Previous studies have shown that ericoid mycorrhizal fungi possess both symbiotic and saprotrophic traits and encode laccase genes capable of degrading phenolic compounds [12,21,61,62,63,64,65], collectively contributing to accelerated decomposition of phenolic substances in *Sphagnum*.

### 4.3. Microbial Associations Involved in S. palustre Decomposition Are Regulated by the C:N Ratio and pH

In this study, the C:N ratio increased significantly from W to Z and was positively correlated with the abundances of Serendipitaceae and Bryobacteraceae (Figure 3g and Figure 6c,d). Serendipitaceae possess numerous genes associated with saprotrophic activity [43], while Bryobacteraceae can degrade plant cellulose [45], suggesting that these two microbial taxa may act synergistically in the decomposition of *S. palustre*.

Moreover, pH is a key environmental factor influencing microbial community structure and may indirectly affect *S. palustre* decomposition [66]. In this study, *S. palustre* pH increased significantly from W to G and was positively correlated with the abundance of Archaeorhizomycetaceae (Figure 3h and Figure 6d), indicating that the pH conditions of the peatland are more favorable for the growth and metabolic activity of this fungus. Archaeorhizomycetaceae is a key saprotrophic fungus in shrub-dominated peatlands, capable of utilizing glucose and cellulose, and is therefore likely involved in the decomposition of *S. palustris* [3,67].

### 4.4. Limitations

We acknowledge that the lack of within-site replication in the *S. palustre*-dominated peatland (control site) limits the statistical power of the control. Although our results indicate that fine root invasion of *R. auriculatum-S. palustre* peatland increases the degree of *S. palustre* decomposition and alters the trophic modes of bacteria and fungi in decomposing *S. palustre*, we recognize that the absence of replication in the control site restricts the generalizability of this conclusion. To address this limitation, we will increase the number of sample replicates in the control *S. palustre* peatland in future studies. Furthermore, this study focused exclusively on the *R. auriculatum*–*S. palustre* peatland. Whether similar conclusions can be drawn for *Sphagnum* peatlands invaded by other Ericaceae plants remains to be investigated in future research.

## 5. Conclusions

Our study demonstrates that encroachment of *R. auriculatum* (Sapling) fine roots accelerates the decomposition of *S. palustre*. This acceleration is associated with increased relative abundances and altered trophic modes of bacterial and fungal communities involved in decomposition, Notably, increased relative abundances were found for chemoheterotrophic and aerobic chemoheterotrophic metabolic types in bacteria and for fungal trophic modes, including bryophyte parasite-ectomycorrhizal-ericoid mycorrhizal-undefined saprotroph-wood saprotroph, as well as changes in enzyme activities and physicochemical properties (TN, C:N, and pH). Furthermore, enhanced decomposition leads to greater losses of phenolic compounds, including cinnamic acids and tannins. Collectively, these findings indicate that *R. auriculatum* invasion drives trophic mode restructuring of microbial communities and promotes the degradation of *S. palustre* and its hyaline cells. This finding may affect the carbon sink function of subtropical subalpine peatlands and alter nutrient cycling dynamics. Given that peatlands store approximately one-third of the global terrestrial soil organic carbon, shrub encroachment-accelerated *Sphagnum* decomposition may exacerbate organic carbon release from global peatlands. Future studies are recommended to employ isotope tracing techniques to detect the fate of carbon during the *Sphagnum* decomposition process.

## Data Availability

The data supporting the findings of this study are available from the corresponding author upon reasonable request.

## Supplementary Materials

The following supporting information can be downloaded at: https://www.mdpi.com/article/10.3390/microorganisms14051141/s1, Figure S1. Analysis of bacterial and fungal diversity in *Sphagnum palustre* and fine roots of *Rhododendron auriculatum*; Figure S2. Dominant bacterial and fungal taxa in *S. palustre*-dominated peatland; Figure S3. Fungal families with increased relative abundance in the invaded fine roots of *R. auriculatum*; Figure S4. Bacterial and fungal functional groups in the *R. auriculatum*-*S. palustre* peatland; Figure S5. Differential metabolite analysis of phenols, cinnamic acid, and tannins in *Sphagnum*; Figure S6. Shared differential metabolites among D vs. W, Z vs. W, and G vs. W; Figure S7. Structural changes during *S. palustre* decomposition in *S. palustre* -dominated peatland; Table S1. Bacterial total number of reads and read length after quality checking and normalization; Table S2. Fungal total number of reads and read length after quality checking and normalization; Table S3. Assigned numbers of phyla, genera and ASVs in fine roots of *Rhododendron auriculatum* and in *Sphagnum palustre* across different decomposition stages; Table S4. Differential metabolites among *S. palustre* at different decomposition levels were screened based on variable importance in projection (VIP) > 1 and *p* < 0.05.

## References

[1] Halsey L.A., Vitt D.H., Gignac L.D. (2000). *Sphagnum*-Dominated Peatlands in North America since the Last Glacial Maximum: Their Occurrence and Extent. Bryologist.

[2] Yusup S., Sundberg S., Ooi M.K.J., Zhang M., Sun Z., Rydin H., Wang M., Feng L., Chen X., Bu Z.-J. (2023). Smoke Promotes Germination of Peatland Bryophyte Spores. J. Exp. Bot..

[3] Xue W., Ma H., Deng K., Qiu P., Liu J., Xiang M., Tian J., Liu X. (2025). Substrates-Mediated Microbes Mitigate Carbon Loss in Shrub Peatlands. J. Plant Ecol..

[4] Ma L., Nawaz M.N., Xu Y., Chen X., Liu D., Lu X., Wang H. (2025). Sphagnum Microhabitats Differentiate the Nitrogen-Fixing Microbial Communities in the Dajiuhu Peatland, Central China: Significance of Methane Oxidation for Nitrogen Fixation. Appl. Soil Ecol..

[5] Wang H., Wu L., Xue D., Liu X., Hong L., Mou L., Li X. (2020). Distribution and Environmental Characteristics of *Sphagnum* Peat Bogs in Taishanmiao in Enshi City, Hubei Province. Wetl. Sci..

[6] Chen X., Bu Z., Wang S., Li H., Zhao H. (2009). Niches of Seven Bryophyte Species in Hani Peat Land of Changbai Mountains. J. Appl. Ecol..

[7] Beike A.K., Spagnuolo V., Lüth V., Steinhart F., Ramos-Gómez J., Krebs M., Adamo P., Rey-Asensio A.I., Angel Fernández J., Giordano S. (2015). Clonal in Vitro Propagation of Peat Mosses (*Sphagnum* L.) as Novel Green Resources for Basic and Applied Research. Plant Cell Tissue Organ Cult. (PCTOC).

[8] Rydin H., Jeglum J.K. (2013). The Biology of Peatlands.

[9] Adams D.G., Duggan P.S. (2008). Cyanobacteria-Bryophyte Symbioses. J. Exp. Bot..

[10] Bragina A., Berg C., Cardinale M., Shcherbakov A., Chebotar V., Berg G. (2012). *Sphagnum* Mosses Harbour Highly Specific Bacterial Diversity during Their Whole Lifecycle. ISME J..

[11] Pérez-Rodríguez M., Alten A., Miler M., Kaal J. (2025). Explicit Microrelief-Controlled Decoupling of Initial Aerobic Decay and Leaching (in Hummocks) and Anaerobic Decay (in Hollows) in Surface Layers of a *Sphagnum*-Dominated Peatland. J. Anal. Appl. Pyrolysis.

[12] Rice A.V., Tsuneda A., Currah R.S. (2006). In Vitro Decomposition of *Sphagnum* by Some Microfungi Resembles White Rot of Wood: Decomposition of *Sphagnum* by Microfungi Resembles White Rot of Wood. Fems Microbiol. Ecol..

[13] Rudolph H., Samland J. (1985). Occurrence and Metabolism of *Sphagnum* Acid in the Cell Walls of Bryophytes. Phytochemistry.

[14] Zhu R. (2022). Peat Mosses (*Sphagnum*): Ecologically, Economically, and Scientifically Important Group of Carbon Sequestration Plants. Chin. Bull. Bot..

[15] Dial R.J., Maher C.T., Hewitt R.E., Sullivan P.F. (2022). Sufficient Conditions for Rapid Range Expansion of a Boreal Conifer. Nature.

[16] Hupperts S.F., Islam K.S., Gundale M.J., Kardol P., Sundqvist M.K. (2024). Warming Influences Carbon and Nitrogen Assimilation between a Widespread Ericaceous Shrub and Root-Associated Fungi. New Phytol..

[17] Defrenne C.E., Moore J.A.M., Tucker C.L., Lamit L.J., Kane E.S., Kolka R.K., Chimner R.A., Keller J.K., Lilleskov E.A. (2023). Peat Loss Collocates with a Threshold in Plant–Mycorrhizal Associations in Drained Peatlands Encroached by Trees. New Phytol..

[18] Wang X., Zhang P., Zhang Y., Wang J., He P., Zheng L., Ling N., Wang Z. (2025). Grassland Shrub Encroachment Generates Divergent Contributions of Plant- and Microbial-Derived Carbon to Soil Organic Carbon. Plant Soil.

[19] Buttler A., Bragazza L., Laggoun-Défarge F., Gogo S., Toussaint M.-L., Lamentowicz M., Chojnicki B.H., Słowiński M., Słowińska S., Zielińska M. (2023). Ericoid Shrub Encroachment Shifts Aboveground–Belowground Linkages in Three Peatlands across Europe and Western Siberia. Glob. Change Biol..

[20] St James A.R., Yavitt J.B., Zinder S.H., Richardson R.E. (2021). Linking Microbial *Sphagnum* Degradation and Acetate Mineralization in Acidic Peat Bogs: From Global Insights to a Genome-Centric Case Study. ISME J..

[21] Martino E., Morin E., Grelet G., Kuo A., Kohler A., Daghino S., Barry K.W., Cichocki N., Clum A., Dockter R.B. (2018). Comparative Genomics and Transcriptomics Depict Ericoid Mycorrhizal Fungi as Versatile Saprotrophs and Plant Mutualists. New Phytol..

[22] Kalam S., Basu A., Ahmad I., Sayyed R.Z., El-Enshasy H.A., Dailin D.J., Suriani N.L. (2020). Recent Understanding of Soil Acidobacteria and Their Ecological Significance: A Critical Review. Front. Microbiol..

[23] Orellana L.H., Francis T.B., Ferraro M., Hehemann J.-H., Fuchs B.M., Amann R.I. (2022). Verrucomicrobiota Are Specialist Consumers of Sulfated Methyl Pentoses during Diatom Blooms. ISME J..

[24] Rakitin A.L., Kulichevskaya I.S., Beletsky A.V., Mardanov A.V., Dedysh S.N., Ravin N.V. (2024). Verrucomicrobia of the Family Chthoniobacteraceae Participate in Xylan Degradation in Boreal Peat Soils. Microorganisms.

[25] Lisov A., Belova O., Zavarzina A., Konstantinov A., Leontievsky A. (2021). The Role of Laccase from Zygomycetous Fungus Mortierella Elasson in Humic Acids Degradation. Agronomy.

[26] Ju M., Zhang Q., Wang R., Yan S., Li Z., Li P., Gu P. (2022). Correlation in Endophytic Fungi Community Diversity and Bioactive Compounds of Sophora Alopecuroides. Front. Microbiol..

[27] Yang X., Chen H., Wu L., Guo X., Xue D. (2025). Diversity and Correlation Analysis of Microbiomes and Metabolites of *Sphagnum Palustre* in Various Microhabitats. BMC Plant Biol..

[28] Deng Y., Huang H., Lei F., Fu S., Zou K., Zhang S., Liu X., Jiang L., Liu H., Miao B. (2021). Endophytic Bacterial Communities of Ginkgo Biloba Leaves during Leaf Developmental Period. Front. Microbiol..

[29] Cui J., Gong Y., Vijayakumar V., Zhang G., Wang M. (2019). Correlation in Chemical Metabolome and Endophytic Mycobiome in Cynomorium Songaricum from Different Desert Locations in China. J. Agric. Food Chem..

[30] Stierle A., Strobel G., Stierle D. (1993). Taxol and Taxane Production by *Taxomyces andreanae*, an Endophytic Fungus of Pacific Yew. Science.

[31] Qin D., You C., Lan W., Wang Y., Yu B., Peng Y., Xu J., Dong J. (2023). Microbial Assemblages of Schisandraceae Plants and the Correlations between Endophytic Species and the Accumulation of Secondary Metabolites. Plant Soil.

[32] Karimi B., Terrat S., Dequiedt S., Saby N.P.A., Horrigue W., Lelièvre M., Nowak V., Jolivet C., Arrouays D., Wincker P. (2018). Biogeography of Soil Bacteria and Archaea across France. Sci. Adv..

[33] Nguyen N.H., Song Z., Bates S.T., Branco S., Tedersoo L., Menke J., Schilling J.S., Kennedy P.G. (2016). FUNGuild: An Open Annotation Tool for Parsing Fungal Community Datasets by Ecological Guild. Fungal Ecol..

[34] Louca S., Parfrey L.W., Doebeli M. (2016). Decoupling Function and Taxonomy in the Global Ocean Microbiome. Science.

[35] Eivazi F., Tabatabai M.A. (1988). Glucosidases and Galactosidases in Soils. Soil Biol. Biochem..

[36] Li Y., Geng Y., Zhou H., Yang Y. (2026). Comparison of Soil Acid Phosphatase Activity Determined by Different Methods. Chin. J. Eco-Agric..

[37] Eichlerová I., Šnajdr J., Baldrian P. (2012). Laccase Activity in Soils: Considerations for the Measurement of Enzyme Activity. Chemosphere.

[38] Zeng W., Yang Z., Huang Y., Gu Y., Tao J., Liu Y., Xie P., Cai H., Yin H. (2022). Response of Soil Bacterial Community Structure and Co-Occurrence Network Topology Properties to Soil Physicochemical Properties in Long-Term Continuous Cropping Farmland. Acta Microbiol. Sin..

[39] Zhang L., Li M., Zhan L., Lu X., Liang L. (2015). Plasma Metabolomic Profiling of Patients with Diabetes-Associated Cognitive Decline. PLoS ONE.

[40] Li C., Zhang J., Wu R., Liu Y., Hu X., Yan Y., Ling X. (2019). A Novel Strategy for Rapidly and Accurately Screening Biomarkers Based on Ultraperformance Liquid Chromatography-Mass Spectrometry Metabolomics Data. Anal. Chim. Acta.

[41] Bougoure D.S., Cairney J.W.G. (2005). Assemblages of Ericoid Mycorrhizal and Other Root-associated Fungi from *Epacris pulchella* (Ericaceae) as Determined by Culturing and Direct DNA Extraction from Roots. Environ. Microbiol..

[42] Allen T.R., Millar T., Berch S.M., Berbee M.L. (2003). Culturing and Direct DNA Extraction Find Different Fungi from the Same Ericoid Mycorrhizal Roots. New Phytol..

[43] Weiß M., Waller F., Zuccaro A., Selosse M.-A. (2016). Sebacinales—One Thousand and One Interactions with Land Plants. New Phytol..

[44] Campbell B.J., Rosenberg E., DeLong E.F., Lory S., Stackebrandt E., Thompson F. (2014). The Family Acidobacteriaceae. The Prokaryotes: Other Major Lineages of Bacteria and the Archaea.

[45] Dedysh S.N., Trujillo M.E., Dedysh S., DeVos P., Hedlund B., Kämpfer P., Rainey F.A., Whitman W.B. (2019). *Bryobacteraceae*. Bergey’s Manual of Systematics of Archaea and Bacteria.

[46] Dedysh S.N., Ivanova A.A., Trujillo M.E., Dedysh S., DeVos P., Hedlund B., Kämpfer P., Rainey F.A., Whitman W.B. (2025). *Terriglobales*. Bergey’s Manual of Systematics of Archaea and Bacteria.

[47] Oren A., Rosenberg E., DeLong E.F., Lory S., Stackebrandt E., Thompson F. (2014). The Family *Xanthobacteraceae*. The Prokaryotes: Alphaproteobacteria and Betaproteobacteria.

[48] Chung E.J., Park T.S., Jeon C.O., Chung Y.R. (2012). *Chitinophaga oryziterrae* Sp. Nov., Isolated from the Rhizosphere Soil of Rice (*Oryza sativa* L.). Int. J. Syst. Evol. Microbiol..

[49] Rosenberg E., Rosenberg E., DeLong E.F., Lory S., Stackebrandt E., Thompson F. (2014). The Family *Chitinophagaceae*. The Prokaryotes: Other Major Lineages of Bacteria and the Archaea.

[50] Bautista-Cruz A., Aquino-Bolaños T., Hernández-Canseco J., Quiñones-Aguilar E.E. (2024). Cellulolytic Aerobic Bacteria Isolated from Agricultural and Forest Soils: An Overview. Biology.

[51] Trujillo-Cabrera Y., Ponce-Mendoza A., Vásquez-Murrieta M.S., Rivera-Orduña F.N., Wang E.T. (2013). Diverse Cellulolytic Bacteria Isolated from the High Humus, Alkaline-Saline Chinampa Soils. Ann. Microbiol..

[52] Leifheit E.F., Camenzind T., Lehmann A., Andrade-Linares D.R., Fussan M., Westhusen S., Wineberger T.M., Rillig M.C. (2024). Fungal Traits Help to Understand the Decomposition of Simple and Complex Plant Litter. FEMS Microbiol. Ecol..

[53] Niego A.G.T., Rapior S., Thongklang N., Raspé O., Hyde K.D., Mortimer P. (2023). Reviewing the Contributions of Macrofungi to Forest Ecosystem Processes and Services. Fungal Biol. Rev..

[54] Ge H., Liu X., Lu D., Yang Z., Li H. (2024). Degradation of Pyrene by *Xanthobacteraceae* Bacterium Strain S3 Isolated from the Rhizosphere Sediment of Vallisneria Natans: Active Conditions, Metabolite Identification, and Proposed Pathways. Environ. Sci. Pollut. Res..

[55] Nagy L.G., Branco S., Floudas D., Hibbett D.S., Lofgren L., Martin F., Merényi Z., Plett J.M., Pringle A., Varga T. (2026). The Biodiversity, Genomics, Ecology and Evolution of Mushroom-Forming Fungi. Nat. Rev. Biodivers..

[56] Kues U., Ruhl M. (2011). Multiple Multi-Copper Oxidase Gene Families in Basidiomycetes—What For?. Curr. Genom..

[57] Zhou M., Fakayode O.A., Ren M., Li H., Liang J., Yagoub A.E.A., Fan Z., Zhou C. (2023). Laccase-Catalyzed Lignin Depolymerization in Deep Eutectic Solvents: Challenges and Prospects. Bioresour. Bioprocess..

[58] Almajanni Y., Amiri H., Taheri-Kafrani A. (2025). Efficient and Cost-Effective Biodegradation of Phenolic Compounds in Lignocellulosic Biomasses Using Laccase Immobilized onto Magnetically Recoverable Nanocellulose-Functionalized Iron-Oxide Nanoparticles. Ind. Crops Prod..

[59] (2006). Phenol 2-Monooxygenase. Class 1 Oxidoreductases XI: EC 1.14.11–1.14.14.

[60] Luo Z., Mao X., Peng M., Huang C., Liang J., Xiao Y., Abubakar Y.S., Zheng W., Xiong Y., Wang Z. (2025). The Phenol-2-Monooxygenase FgPhm1 Regulates DON Synthesis, Pathogenicity and Environmental Stress Response in *Fusarium graminearum*. Virulence.

[61] Rice A.V., Currah R.S. (2005). *Oidiodendron*: A Survey of the Named Species and Related Anamorphs of *Myxotrichum*. Stud. Mycol..

[62] Rice A.V., Currah R.S., Schulz B.J.E., Boyle C.J.C., Sieber T.N. (2006). Oidiodendron Maius: Saprobe in *Sphagnum* Peat, Mutualist in Ericaceous Roots?. Microbial Root Endophytes.

[63] Bruyant P., Moënne-Loccoz Y., Almario J. (2024). Root-Associated Helotiales Fungi: Overlooked Players in Plant Nutrition. Soil Biol. Biochem..

[64] Ward E.B., Duguid M.C., Kuebbing S.E., Lendemer J.C., Bradford M.A. (2022). The Functional Role of Ericoid Mycorrhizal Plants and Fungi on Carbon and Nitrogen Dynamics in Forests. New Phytol..

[65] Burke R., Cairney J. (2002). Laccases and Other Polyphenol Oxidases in Ecto- and Ericoid Mycorrhizal Fungi. Mycorrhiza.

[66] Li X., Chen D., Carrión V.J., Revillini D., Yin S., Dong Y., Zhang T., Wang X., Delgado-Baquerizo M. (2023). Acidification Suppresses the Natural Capacity of Soil Microbiome to Fight Pathogenic Fusarium Infections. Nat. Commun..

[67] Rosling A., Cox F., Cruz-Martinez K., Ihrmark K., Grelet G.-A., Lindahl B.D., Menkis A., James T.Y. (2011). Archaeorhizomycetes: Unearthing an Ancient Class of Ubiquitous Soil Fungi. Science.

