# Supplementary material for: Combined Microbiome and Metabolomic Analyses Reveal That Fine-Root Invasion of Rhododendron auriculatum Sapling Enhances Microbial Decomposition of Sphagnum palustre L
Source: Microorganisms. 2026 May 17;14(5):1141. doi: 10.3390/microorganisms14051141 (PMC13209657; doi:10.3390/microorganisms14051141)
Supplement: Supplementary file 1 [file microorganisms-14-01141-s001.zip › microorganisms-4282823-supplementary.pdf]

Supporting Information

Article title: Combined Microbiome and Metabolomic Analyses Reveal That Fine-Root Invasion of *Rhododendron auriculatum* Sapling Enhances Microbial Decomposition of *Sphagnum palustre* L.

Authors:Qiuxia Xiang, Guijun Bu *, Xiaorong Tang, Changwu Shi, Bing Xiong, Lin Wu and Jia Xiong

Hubei Key Laboratory of Biological Resources Protection and Utilization (Hubei Minzu University), Enshi 445000, China

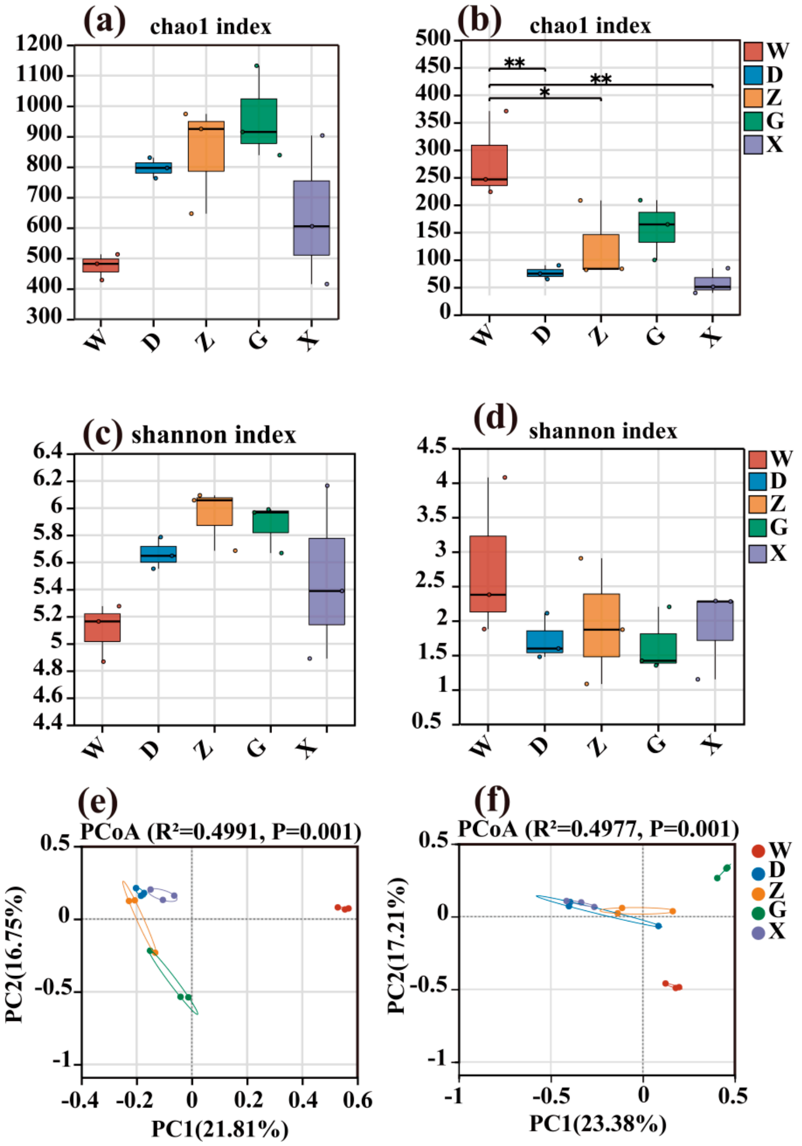

Figure S1. Analysis of bacterial and fungal diversity in *Sphagnum palustre* and fine roots of *Rhododendron auriculatum*. (a) and (c) represent the Chao1 index and Shannon index of bacteria, respectively; (b) and (d) represent the Chao1 index and Shannon index of fungi, respectively; (e) shows bacterial β-diversity, and (f) shows fungal β-diversity.

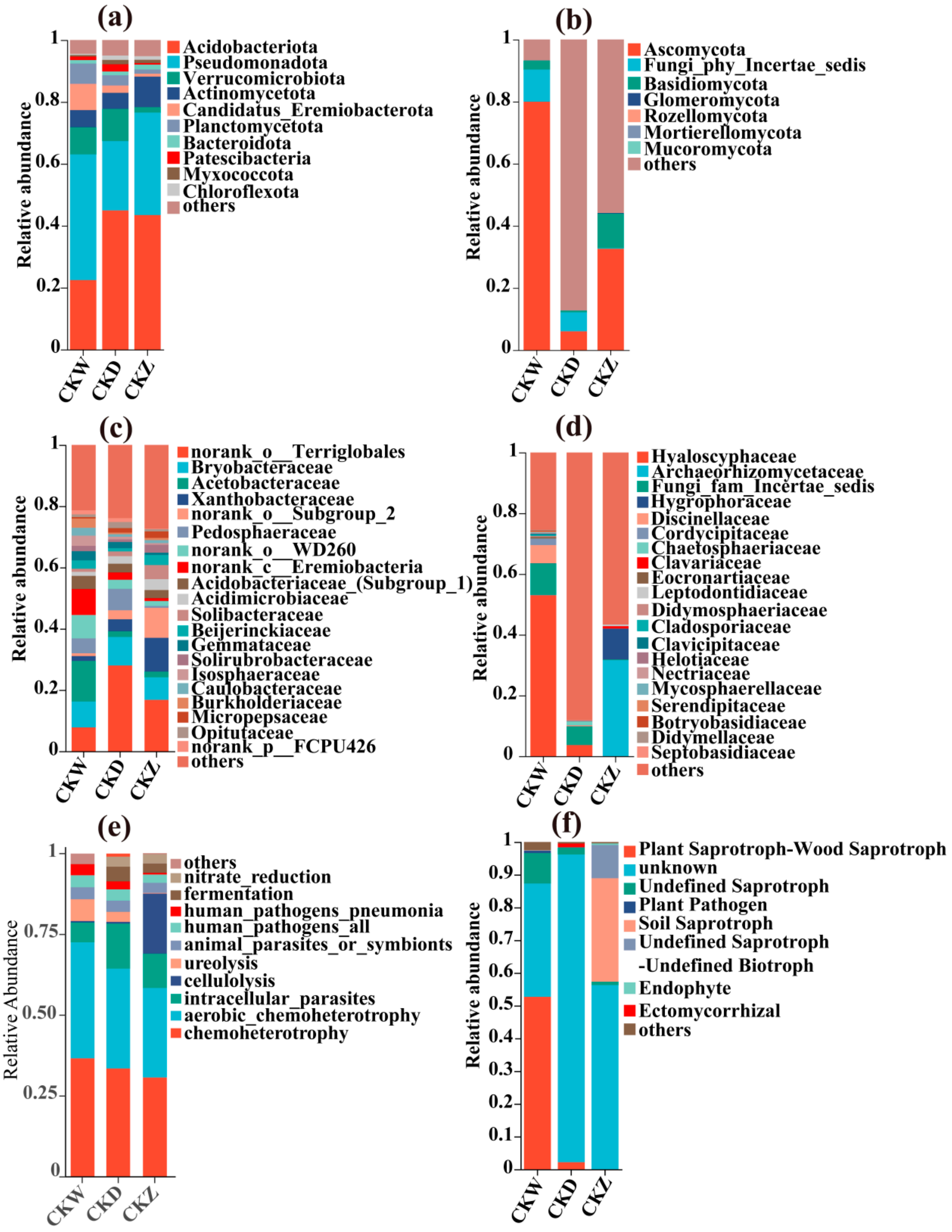

Figure S2. Dominant bacterial and fungal taxa in *S. palustre*-dominated peatland. (a) bacterial taxa at the phylum level, (b) fungal taxa at the phylum level, (c) bacterial taxa at the family level, (d) fungal taxa at the family level, (e) bacterial functional groups, (f) fungal functional groups.

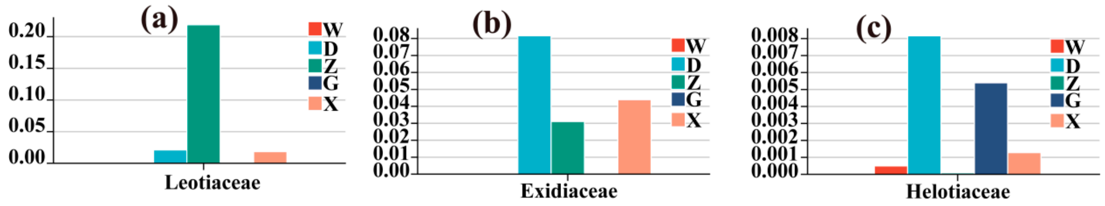

Figure S3 Fungal families with increased relative abundance in the invaded fine roots of *R. auriculatum* (D). (a) Leotiaceae, (b) Exidiaceae, (c) Helotiaceae.

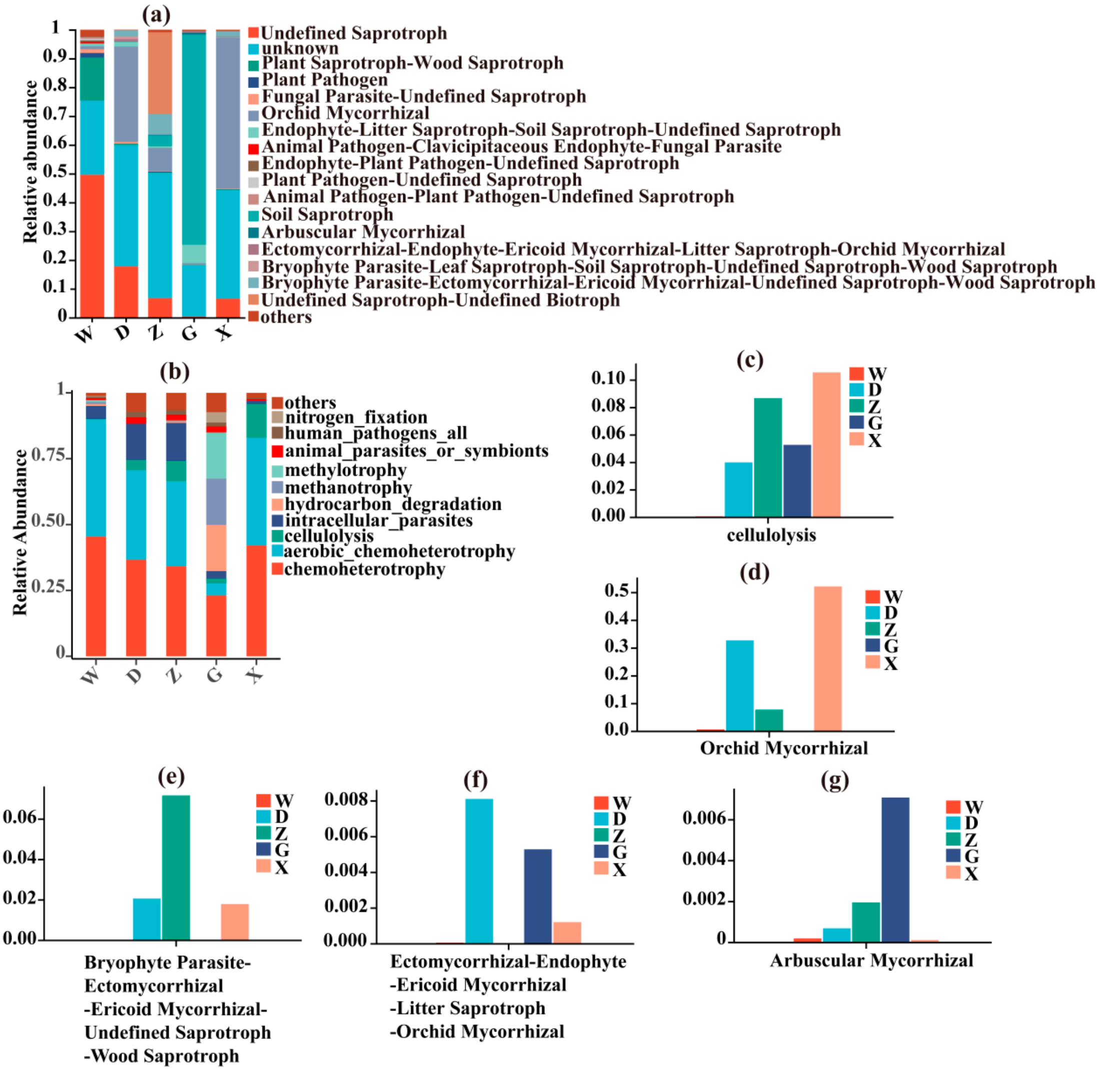

Figure S4. Bacterial and fungal functional groups in the *R. auriculatum-S. palustre* peatland. (a) Fungal functional groups, (b) bacterial functional groups, (c) relative abundance of cellulolysis, (d) relative abundance of orchid mycorrhizal fungi, (e) relative abundance of bryophyte parasite–ectomycorrhizal–ericoid mycorrhizal–undefined saprotroph–wood saprotroph, (f) relative abundance of ectomycorrhizal–endophyte–ericoid mycorrhizal–litter saprotroph–orchid mycorrhizal, and (g) relative abundance of arbuscular mycorrhizal fungi.

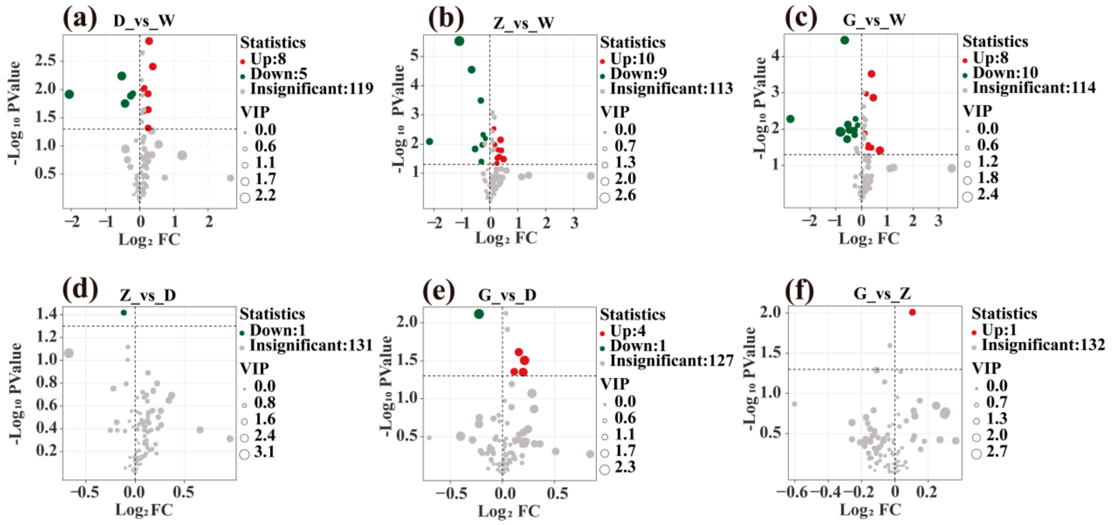

Figure S5. Differential metabolite analysis of phenols, cinnamic acid, and tannins in *Sphagnum*. (a) differential metabolites in D vs. W, (b) differential metabolites in Z vs. W, (c) differential metabolites in G vs. W, (d) differential metabolites in Z vs. D, (e) differential metabolites in G vs. D, and (f) differential metabolites in Z vs. G.

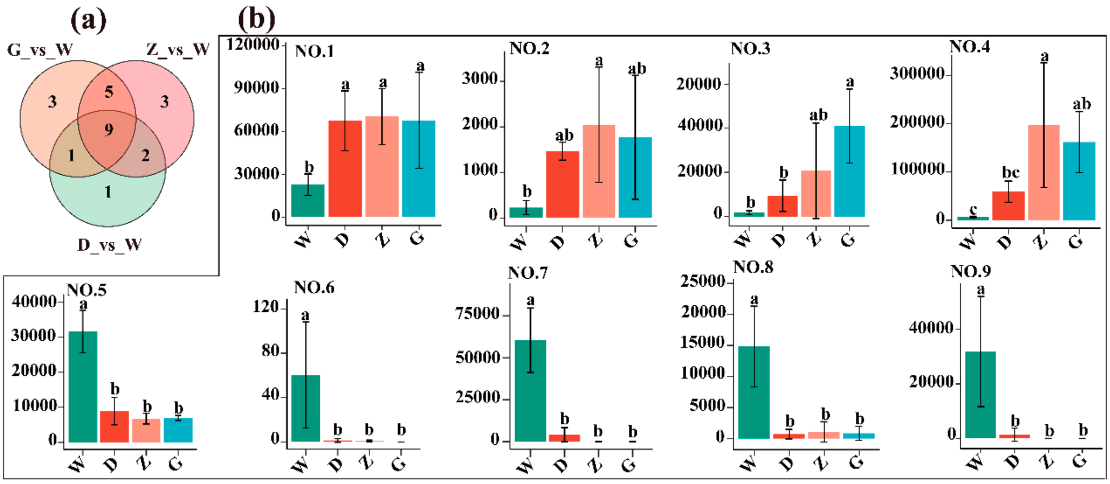

Figure S6. Shared differential metabolites among D vs. W, Z vs. W, and G vs. W. (a) Venn diagram of D vs. W, Z vs. W, and G vs. W; (b) NO.1: Carthamone, NO.2: Coumarinic acid, NO.3: N-[2-(3,4-Dihydroxyphenyl)ethyl]icosa-5,8,11,14-tetraenamide, NO.4: 5-Tricosyl-1,3-benzenediol, NO.5: Octopamine, NO.6: Theasinensin C, NO.7: 2-Hydroxy-3-methyl-4H-pyran-4-one O-(6E-cinnamoyl-β-D-glucoside), NO.8: 4-Heptyloxyphenol, NO.9: Ellagic acid acetyl-xyloside.

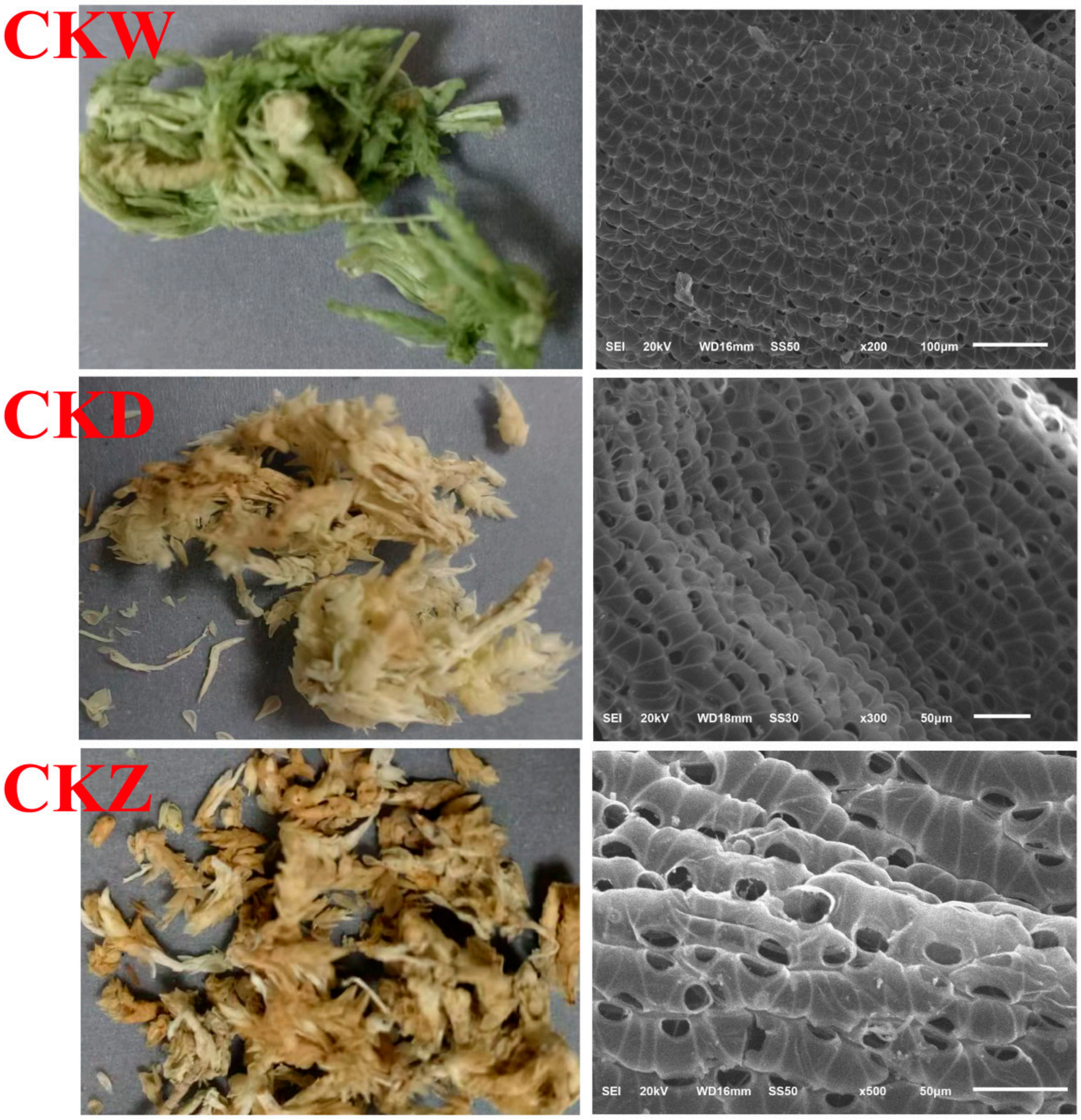

Figure S7. Structural changes during *S. palustre* decomposition in *S. palustre* -dominated peatland

Table S1 Bacterial total number of reads and read length after quality checking and normalization.

Sample	Sequence numbers	Base sequence num(bp)	Mean sequence length(bp)	Min sequence length(bp)	Max sequence length(bp)
W1	76868	31415590	408.695296	262	523
W2	46928	19239033	409.969166	239	432
W3	64827	26636457	410.885233	262	474
D1	82874	34201767	412.695984	239	527
D2	67426	27866922	413.296384	239	459
D3	76652	31598961	412.239224	297	442
Z1	70398	29072080	412.967414	214	438
Z2	72725	30059068	413.325101	245	516
Z3	56999	23424307	410.959964	233	453
G1	73099	30129623	412.175584	216	461
G2	96201	39679104	412.460411	239	516
G3	68240	28074356	411.406155	258	452
XG1	75603	31215238	412.883589	291	539
XG2	67336	27807846	412.971457	203	516
XG3	60257	25037727	415.515658	205	440
CKW	77891	32047394	411.438985	261	437
CKD	71107	29293762	411.967345	262	440
CKZ	61595	25372859	411.930498	239	483

Table S2 Fungal total number of reads and read length after quality checking and normalization.

Sample	Sequence numbers	Base sequence num(bp)	Mean sequence length(bp)	Min sequence length(bp)	Max sequence length(bp)
W1	68021	16923335	248.79574	140	381
W2	60704	14388750	237.031332	139	528
W3	54388	12811919	235.56518	139	534
D1	46414	12838226	276.602448	99	392
D2	41441	10621226	256.297531	155	525
D3	50561	13176572	260.607425	67	377
Z1	60636	15289149	252.146398	118	527
Z2	50306	13379511	265.962529	140	351
Z3	68322	16229613	237.54593	136	317
G1	75678	14503144	191.642802	125	288
G2	81792	16059513	196.345767	143	538
G3	75935	15670357	206.365405	117	538
XG1	46861	12605974	269.007789	121	331
XG2	46669	12396990	265.636504	139	373
XG3	44193	12085649	273.474283	176	336
CKW	60729	14153650	233.062458	139	536
CKD	63292	13648668	215.646022	74	335
CKZ	149684	28247207	188.712267	77	518

Table S3 Assigned numbers of phyla, genera and ASVs in fine roots of *Rhododendron auriculatum* and in *Sphagnum palustre* across different decomposition stages.

Bacteria	Phylum	Family	ASV	Fungi	Phylum	Family	ASV
W	21	138	985	W	9	162	691
D	21	172	1933	D	9	174	192
Z	25	190	2137	Z	10	187	307
G	34	211	2257	G	9	199	365
X	24	184	1627	X	7	202	146
CKW	21	117	620	CKW	6	89	199
CKD	20	138	686	CKD	7	31	87
CKZ	23	135	760	CKZ	7	33	105
Total	35	297	7898	Total	10	202	1425

Table S4. Differential metabolites among *S. palustre* at different decomposition levels were screened based on variable importance in projection (VIP) > 1 and $p < 0.05$.

Metabolite	m/z	Retention time	Mode	Mass Error (ppm)	W1	W2	W3	D1	D2	D3	Z1	Z2	Z3	G1	G2	G3
Phloroglucinol	127.04	0.67	pos	-2.87	405219.83	182726.03	202721.75	188975.74	276062.59	74277.81	77984.17	74414.27	69719.81	75300.22	79289.88	78289.96
Octopamine	136.08	1.00	pos	-1.94	33432.72	36584.35	24863.04	4420.27	10732.32	11578.12	5396.01	8419.25	6442.67	6132.30	7327.64	7406.15
Osmanthuside B	593.22	4.17	pos	-4.31	229815.36	197430.82	229283.16	172185.23	2099.85	2131.63	25413.63	18254.11	14167.11	14775.23	0.00	12606.90
Vanillin	153.05	4.25	pos	-2.19	531.28	944.17	1228.60	2577.06	679.27	212.22	2773.81	1328.44	253.12	4450.44	2538.68	5641.07
4-Heptyloxyphenol	173.13	5.98	pos	-2.16	12242.66	9997.18	22232.93	1529.99	0.00	618.85	2969.61	0.00	336.08	2085.38	0.00	439.11
1,26-Dicaffeoylhexacosanediol	723.48	6.37	pos	0.97	376072.42	219606.07	273354.81	408449.65	991025.45	25131.88	54219.32	9535.16	192132.62	87181.65	31650.18	14859.87
Norfenefrine	118.06	6.00	pos	-2.17	934.77	222.30	326.96	2273.06	407.39	2225.04	2397.25	2148.21	2142.37	1883.40	1700.41	1927.70
Isoetharine	240.16	5.98	pos	-11.52	11729.75	17334.88	5134.19	13868.91	11153.31	9697.61	7483.34	14128.38	10939.93	22572.32	27936.70	17551.92
Osmanthuside A	447.16	3.61	pos	-6.81	0.00	1052.02	2427.54	625.80	933.96	7041.13	5688.36	3601.10	11901.02	6970.50	0.00	7342.08
Theasinensin C	611.14	2.42	pos	-2.51	52.18	111.90	17.04	0.00	3.26	0.53	0.00	1.12	1.46	0.00	0.00	0.00
2-Hydroxy-3-methyl-4H-pyran-4-oneO-(6E-cinnamoyl-b-D-glucoside)	460.16	2.36	pos	-9.20	44301.39	55378.94	81718.69	8394.43	4128.05	0.00	0.00	0.00	0.00	0.00	0.00	0.00

Metabolite	m/z	Retention time	Mode	Mass Error (ppm)	W1	W2	W3	D1	D2	D3	Z1	Z2	Z3	G1	G2	G3
3,4-dihydroxymandelic acid	367.06	1.87	neg	-6.19	39127.37	8904.62	30686.48	35913.71	1767.46	334.19	4222.69	711.47	3429.71	340.91	122.29	2462.90
Isorhamnetin 3-rutinoside-7-glucoside	311.08	3.14	neg	1.05	874120.34	490389.00	1182060.98	273976.44	35512.27	442.77	225.20	0.00	0.00	0.00	223.14	19106.49
Vanilloyl glucose	329.09	3.75	neg	1.09	0.00	5003.16	0.00	44214.44	2517.97	5650.18	39609.47	9261.45	40417.93	28111.72	0.00	21150.32
Sinapic acid	223.06	5.49	neg	0.53	1741.02	1347.25	2039.29	4414.80	2131.42	4455.44	5937.60	3464.28	6933.68	5252.84	3341.57	4046.92
2,4-dinitrophenol	183.00	5.94	neg	1.05	1905.61	2423.85	2316.06	3987.00	4525.02	7130.40	6027.94	6703.74	5400.68	6440.01	7810.07	6103.08
Coumarinic acid	145.03	6.00	neg	0.10	405.45	143.21	128.88	1682.87	1301.79	1415.05	633.91	3071.73	2439.35	450.94	1687.59	3173.77
Carthamone	469.08	6.02	neg	2.48	26717.53	14244.13	27447.27	44352.22	73145.69	85258.37	52184.40	91382.69	67794.76	42740.52	106117.34	54799.89
3,4-Dihydroxyphenylacetic acid	167.04	6.15	neg	0.67	1805.68	30548.05	1112.30	33261.71	21833.54	14152.47	6525.65	12893.33	7175.12	2268.03	5027.93	5059.25
N-[2-(3,4-Dihydroxyphenyl) ethyl]icosa-5,8,11,14-tetraenamide	438.30	6.43	neg	-3.93	2428.01	2001.62	789.53	17229.14	7500.06	3359.54	45618.92	9111.27	7276.15	30593.33	32025.92	60299.45
5-Nonadecyl-1,3-benzenediol	421.33	6.55	neg	2.48	0.00	855.79	1275.34	9356.26	3954.96	7286.84	20104.63	4017.63	7609.58	23468.92	21359.96	17194.76
5-Tricosyl-1,3-benzenediol	477.40	6.83	neg	2.09	6353.61	5830.58	8818.59	84273.17	42639.26	51633.78	261906.33	48640.29	281119.57	177220.41	215929.19	92740.99
Eugenol	373.17	6.08	neg	0.80	5904.86	12247.78	5040.55	10831.43	3156.31	3274.05	14302.37	8189.52	14713.04	33464.48	15499.23	16975.44
Isohomovanillic acid	181.05	6.07	neg	1.02	1995.27	2403.76	1158.25	14595.57	6882.14	6789.02	25195.12	6465.81	5619.35	42570.91	5138.47	4701.48
Ethyl caffeate	207.07	6.02	neg	0.33	45812.74	30153.22	47512.26	64469.83	102229.94	112812.87	81001.83	112774.93	204581.36	57388.15	130048.87	75303.65

Metabolite	m/z	Retention time	Mode	Mass Error (ppm)	W1	W2	W3	D1	D2	D3	Z1	Z2	Z3	G1	G2	G3
Methyl 4-hydroxycinnamate	177.06	6.00	neg	0.80	1805.35	2514.15	1330.67	18486.99	5323.65	8417.55	4736.33	10280.72	28480.84	9009.82	4293.03	46246.61
Ellagic acid acetyl-xyloside	497.03	3.79	neg	-7.72	36588.09	9684.85	49235.18	4032.08	0.00	0.00	0.00	0.00	0.00	0.00	0.00	0.00
